# Warning and active steering rollover prevention control for agricultural wheeled tractor

**DOI:** 10.1371/journal.pone.0280021

**Published:** 2022-12-30

**Authors:** Jie Gao, Chengqiang Yin, Guanhao Yuan

**Affiliations:** 1 School of Machinery and Automation, Weifang University, Weifang, Shandong Province, China; 2 School of Mechanical and Automobile Engineering, Liaocheng University, Liaocheng, Shandong Province, China; Beijing Institute of Technology, CHINA

## Abstract

Tractor rollover is regarded as the most fatal incident in agricultural production, but some of which can be avoided by timely anti-rollover warning and active control. There have been a lot of researches on the tractor rollover model building and rollover protective structure designing but few on the anti-rollover control. The purpose of this study is to develop a cheaper and practical anti-rollover control system based on active steering technique and to prove the efficiency of the proposed scheme for the wheeled tractors. A three-degree-of-freedom rollover dynamics model including automatic steering system is established. A control scheme by adjusting the roll angle to keep the stability based on the adaptive sliding mode control is proposed with the estimated lateral velocity according to the feedback correction principle. Front wheel angle tracking controller is designed adopting internal model control (IMC) theory. Simulation results exhibit that the active anti-rollover control can calculate the stability index in real time and can keep it within the stable range by adjusting the front steering wheel angle. It is prospective for the proposed scheme to provide a valuable reference to reduce tractor rollover accidents.

## Introduction

With the development of agricultural machinery and modern agriculture, more and more agricultural machineries have been applied in hilly and mountainous areas. It is no doubt that tractor is the core power equipment in agricultural production, and its ownership is growing persistently. But it is noteworthy that the tractor rollover accident has become a threat in the agricultural production. Although there is a great progress in the research on the tractor security, rollover is still one of the most dangerous factors in the process of agricultural operation. According to statistics, nearly 50% of the death accidents caused by tractors are from tractor rollover [1]. By analyzing the occurrence of rollover accidents, we can see that the structure of the tractor, operating environment, or improper operation of the driver will give rise to rollover. Therefore, study on anti-rollover of tractor has been an important subject for many years [2]. Consulting the relevant literature, we can conclude that most of the researches focus on the establishment of the tractor rollover model, the design of rollover protective device and the developing of rollover warning system.

It is a complicated work to establish a model to describe the dynamics of tractor rollover, but it is the basis of the study on preventing tractor rollover. Therefore, a large number of experts and scholars are devoted to studying the models that can express really the characteristics of the tractor. Computer simulation is the main method used to establish the tractor model to explore the principle of the tractor rollover. And Newton method or Lagrange are the commonly used theories for building the tractor dynamic models [3]. Dynamic of energy is also the promising method for studying the rollover principle, it is adopted to describe the rollover characteristic of the tractor [4–6]. In view of the complexity of tractor during operations, assumptions have been made when establishing the model of tractor, for instance, tractor was regarded as a rigid body with reversible front, tire deformation was ignored [7], and center of the tractor was regarded as its center of mass [8–10]. For these models, it is difficult to demonstrate dynamic behavior of the tractor for the most of the models because of the too much limitation. Li et al. [11] established a dynamic model according to the actual configuration and motion characteristics of the tractor in three-dimensional coordinate system. Baker et al. [12] proposed a general model for predicting the instability of tractors on slopes, but this model is only suitable for studying the rollover principle of tractors centroid at the front. In [13], the influences of the ground slope angle, the shape and height of obstacles on the lateral overturning and backward rollover were studied based on a 3D simulation model. These approaches were presented mainly based on simulation software but little analysis of the mechanical part of the tractor. As we know, the mechanical properties as well as the interaction conditions will likely to lead to the rollover of the tractor. Therefore, it would be beneficial to develop the dynamic model using the analytical method.

It is well known that the reasonable design of rollover protective structure (ROPS) can potentially decrease the severity of injuries and the death toll in tractor rollover incidents, because it can absorb the energy generated by the weight of the tractor and can provide a safe space in the rollover process. So for many years, ROPS has been one of the hotspots in tractor stability research. The study of ROPS involved many aspects, such as simulation and experiment about the kinematic effect of rollover protection size on tractor, standardization of test procedures for rollover protective structures [14–17].

Rollover protection device installed on the tractor can really alleviate consequences of rollover, but it cannot prevent the tractor from rollover. Therefore, researchers have been focused on developing warning system about the state of tractor in time, or studying on active rollover prevention technology according to co-driving of human-machine intervention idea [18]. Liu et al. [19] designed a tractor rollover detection, emergency reporting and safe driving system based on IOS mobile electronics. Stability is assessed according to the data from the built-in sensors of the equipment. In [20], active steering was adopted to prevent tractor from rollover based on established dynamic steering model and energy-based tractor stability indicator. After that, a coordinate anti-rollover scheme using a single axis momentum flywheel and active steering was presented to adjust the tractor attitude in emergency cases [21]. Recently, the authors put forward a nonlinear time-varying attitude dynamic model and designed an active anti-rollover system [22]. Emergency reporting or rollover alarm system would not avoid all the rollover accidents because of limitations of driver’s experience and reaction ability, but it provides beneficial reference about the judging and expressing of the crucial state of rollover. Anti-rollover based on the active front wheel steering is practical and effective though it can’t cope with all kinds of rollover accidents.

As can be seen that there have been a lot of researches on tractor rollover, but most of the researches focus on the rollover model and the rollover protective structure which cannot fundamentally avoid or decrease the probability of rollover. What’s more, there are few researches on the anti-rollover control for agricultural tractors. The major contribution of this study is that a cheaper and practical anti-rollover warning and control system for wheeled tractor is proposed. An analytical rollover dynamic model including steering system is established and a simplified and effective rollover indicator is derived considering the actual working situation of the tractor. Front wheel steering mode is adopted to maintain the stability by adjusting the roll angle based on the adaptive sliding mode control with lateral velocity estimation according to the feedback correction principle. In order to improve the front wheel angle tracking performance, a simple IMC-PID controller is designed. To demonstrate the effectiveness of the proposed control strategy, a plan path and path tracking controller are devised. During travelling along the plan path, anti-rollover control scheme will exhibit the validity of adjusting the front wheel angle according to the calculated LTR value, at the same time, sliding mode control performance and front wheel tracking performance are also shown in the travelling process. For clear illustration, the paper is organized as follows. Architecture of the rollover prevention system is introduced in Section 2. Models are detailed in Section 3. Section 4 presents the warning and anti-rollover control system design. Effectiveness of the proposed scheme is exhibited in Section 5 by simulation and some conclusions are given in Section 6.

## Architecture of the rollover prevention system

**Fig 1:**
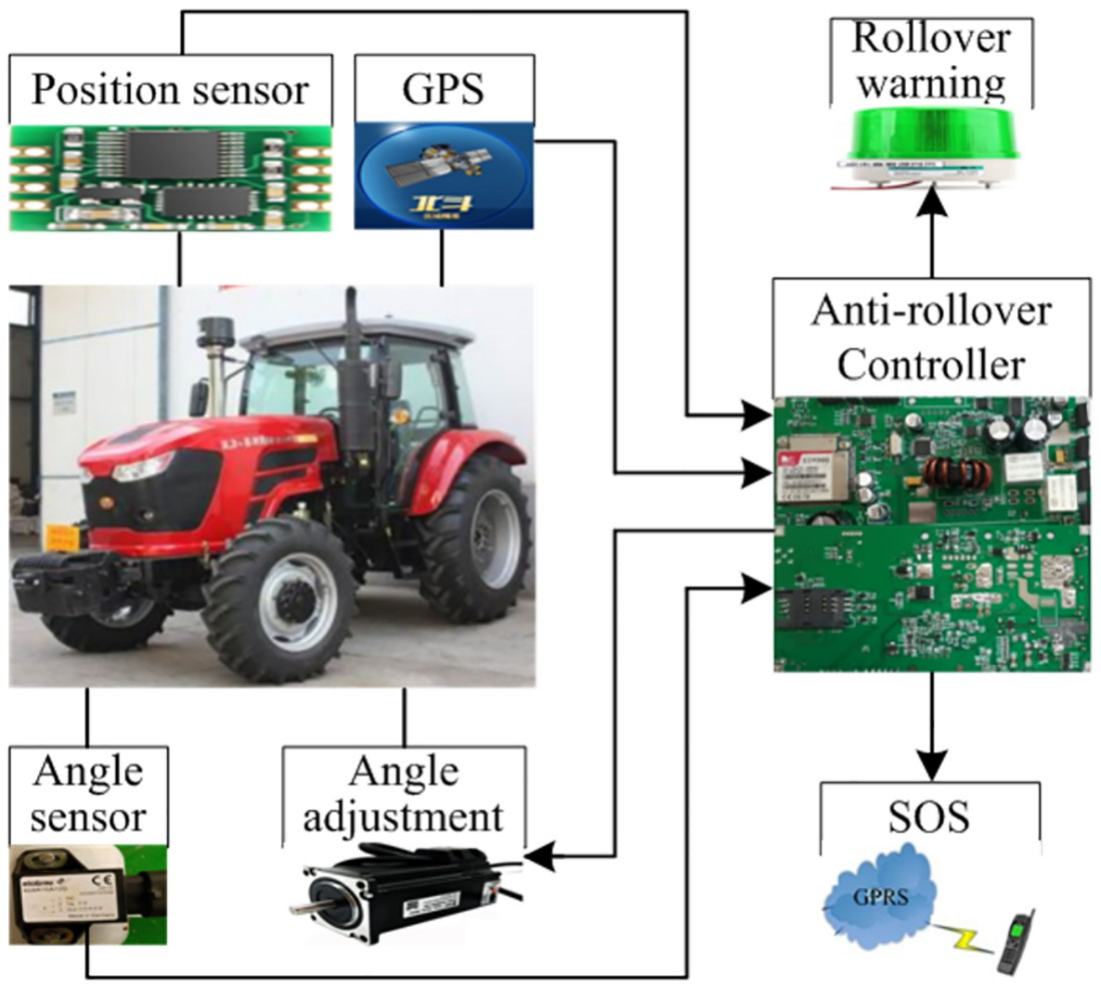
Architecture of rollover prevention system.

As mentioned above, to protect the driver as long as possible, the function of the anti-rollover warning and control system is related to many state parameters such as speed, front wheel angle, yaw speed and roll angle of the tractor required to be obtained in real time. To assess the stability of the tractor, a rollover indicator is designed which can be calculated according to the obtained parameters. The control system compares the calculated indicator with the set value in real time. If the threshold of the warning level is exceeded, anti-rollover warning will be issued to the driver. If the calculated indicator is higher than the set threshold of the control level, the system will automatically activate the rollover prevention control scheme by adjusting the steering wheel of the tractor. At the same time, in order to facilitate the implementation of emergency rescue, the system can automatically obtain the real-time position of the tractor and send the message in the form of information including the position and time to the preset phone number. The architecture of rollover prevention system is designed in Fig 1. In the proposed system, DC motor is selected as the steering driver based on the original hydraulic power to realize the adjustment of the front wheel [23]. And JY61 is adopted as the position sensor to get the yaw speed and roll angle of the tractor by obtaining data including acceleration, angular velocity and angle.

## Model development

### Dynamic model of tractor

It is difficult to establish a model reflecting the dynamic characteristics of tractor accurately because the tractor rollover process is much too complex. Three-degree-of freedom (TDOF) rollover model can reflect the dynamics of tractor rollover and it includes most of the structural parameters of the tractor. A TDOF dynamic model of tractor is presented in this paper. To realize the rollover prevention control by active front wheel steering, some assumptions for the tractor are proposed as follow.

1. The dynamic characteristics of the tractor in the pitching direction are not considered, and the changes in tire characteristics caused by the changes in load and the effect of aligning torque are ignored too.
2. The influence of pitch movement on the tractor is negligible after applied active steering control, and the stiffness and damping of the suspension are regarded as equivalent roll stiffness and equivalent roll damping respectively.
3. The nonlinearity of tire and suspension are ignored, and the difference of steering angle between the left and right wheels during steering is negligible too.

**Fig 2:**
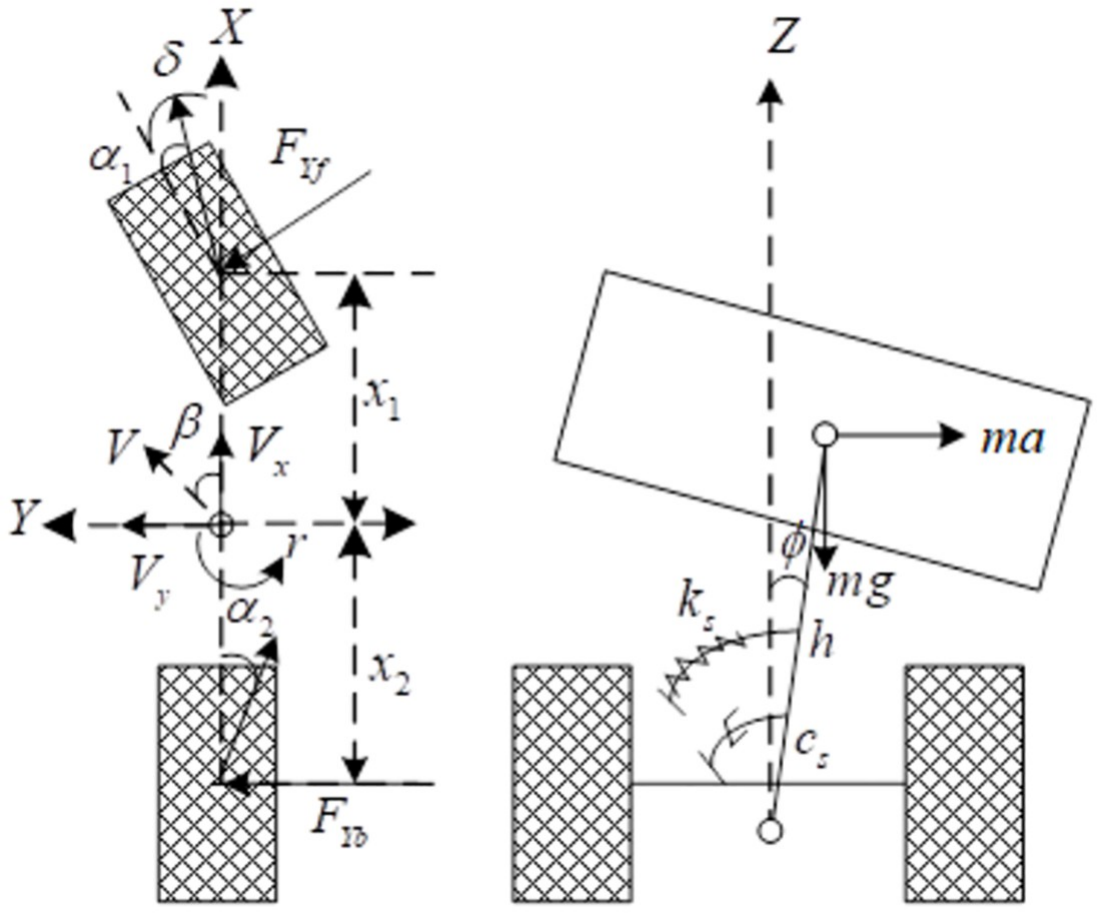
Schematic cosver model.

Based on the assumptions, Fig 2 is used to show the tractor model while it is travelling, where Society of Automotive Engineers (SAE) is used to define the coordinate system of the tractor. The centroid of the tractor is set as the origin of coordinate system, and the tractor driving direction is regarded as the positive direction of the X axis. Y axis is parallel to the ground and perpendicular to the X, and its positive direction is to the left. The positive direction of Z axis is perpendicular to the ground and to up. According to Newton’s second law of motion, the TDOF dynamic model of the tractor can be established as follows.

The equation of motion for the tractor rotating around the X-axis is

$$I_{xeq}\ddot{\phi }=m_{s}ah\;\text{cos}\;\phi +m_{s}gh\;\text{sin}\;\phi -k_{s}\phi -c_{s}\dot{\phi }+M_{\phi }$$ (1)

The equation of motion for the tractor in the Z-axis is

$$I_{z}\ddot{\psi }=x_{1}F_{Y\text{f}}\;\text{cos}\;\delta -x_{2}F_{Yb}$$ (2)

The equation of motion for the tractor in the Y-axis is

$$m({\dot{V}}_{y}+V_{x}r)=F_{Yf}\;\text{cos}\;\delta +F_{Yb}+m_{s}h\ddot{\phi }$$ (3)

Where *α*_1_, *α*_2_ are the sideslip angles of the front and rear axles respectively. *F*_*Yf*_, *F*_*Yb*_ are the lateral force of front and rear tires respectively. *a* is the lateral acceleration of the tractor. *V*_*x*_, *V*_*y*_ are the speed of the tractor along the X axis and Y axis respectively. *r* is the yaw rate of the tractor. *β* is the sideslip angle of the tractor. *ϕ* is the roll angle of the tractor. *δ* is the front wheel angle. *m* is the mass of the tractor, and *m*_*s*_ is sprung mass. *d* is the wheelbase of the tractor, and *h* is the distance from the center of mass to the roll center. *k*_*s*,*Cs*_ are the roll stiffness and roll damping of the suspension respectively. *M*_*ϕ*_ is the additional rolling moment. *x*_1_ is the distance from the center of mass to the front axis, and *x*_2_ is the distance from the center of mass to the rear axis. *k*_1_, *k*_2_ are the cornering stiffness of the front wheel and rear wheel respectively. According to the parallel axis shift theorem, it can be got

$$I_{xeq}=I_{x}+mh^{2}$$ (4)

Where *I*_*x*_ is the moment of inertia of the tractor mass around the X axis; *I*_*xeq*_ is the moment of inertia of the tractor mass around the roll center. In the SAE coordinates, the lateral acceleration of the tractor along the Y axis is

$$a={\dot{V}}_{y}+rV_{x}$$ (5)

Considering the steering angle is generally small, a linear model is used to denote the tire, and the lateral force of the tire can be calculated as follows.


$$F_{Yf}=k_{1}\alpha_{1},F_{Yb}=k_{2}\alpha_{2}$$ (6)

$$\alpha_{1}=-\delta +\frac{V_{y}+rx_{1}}{V_{x}},\alpha_{2}=\frac{V_{y}-rx_{2}}{V_{x}}$$ (7)

Selecting the state variable and output variable as $X={\left[\begin{array}{cccc} V_{y} & r & \phi & \dot{\phi } \end{array}\right]}^{T}$, $Y={\left[\begin{array}{cccc} V_{y} & a & \phi & r \end{array}\right]}^{T}$, and solving Eqs (1)–(3), we can obtain the equation of state space for the TDOF rollover model of the tractor as follows.


$$\left\{\begin{array}{c} \dot{X}=AX+B\delta \\ Y=CX+D\delta \end{array}\right.$$ (8)

$$A=\left[\begin{array}{cccc} -\frac{(k_{1}+k_{2})I_{xeq}}{(mI_{xeq}-m_{s}^{2}h^{2})V_{x}} & -\frac{(x_{1}k_{1}-x_{2}k_{2})I_{xeq}}{(mI_{xeq}-m_{s}^{2}h^{2})V_{x}^{}}-V_{x} & \frac{m_{s}^{2}h^{2}g-m_{s}hk_{s}}{mI_{xeq}-m_{s}^{2}h^{2}} & -\frac{m_{s}hc_{s}}{mI_{xeq}-m_{s}^{2}h^{2}}\\ -\frac{x_{1}k_{1}-x_{2}k_{2}}{V_{x}I_{z}} & -\frac{x_{1}^{2}k_{1}+x_{2}^{2}k_{2}}{I_{z}V_{x}} & 0 & 0\\ 0 & 0 & 0 & 1\\ -\frac{(k_{1}+k_{2})m_{s}h}{(mI_{xeq}-m_{s}^{2}h^{2})V_{x}} & -\frac{(x_{1}k_{1}-x_{2}k_{2})m_{s}h}{(mI_{xeq}-m_{s}^{2}h^{2})V_{x}} & \frac{m(m_{s}gh-k_{s})}{mI_{xeq}-m_{s}^{2}h^{2}} & -\frac{mc_{s}}{mI_{xeq}-m_{s}^{2}h^{2}} \end{array}\right]$$

$$C=\left[\begin{array}{cccc} 1 & 0 & 0 & 0\\ -\frac{(k_{1}+k_{2})I_{xeq}}{(mI_{xeq}-m_{s}^{2}h^{2})V_{x}} & -\frac{(x_{1}k_{1}-x_{2}k_{2})I_{xeq}}{(mI_{xeq}-m_{s}^{2}h^{2})V_{x}} & \frac{m_{s}^{2}h^{2}g-k_{s}}{mI_{xeq}-m_{s}^{2}h^{2}} & -\frac{m_{s}hc_{s}}{mI_{xeq}-m_{s}^{2}h^{2}}\\ 0 & 0 & 1 & 0\\ 0 & 1 & 0 & 0 \end{array}\right]$$

$$B=\left[\begin{array}{c} \frac{k_{1}I_{xeq}}{mI_{xeq}-m_{s}^{2}h^{2}}\\ \frac{x_{1}k_{1}}{I_{z}}\\ 0\\ \frac{m_{s}hk_{1}}{mI_{xeq}-m_{s}^{2}h^{2}} \end{array}\right]D=\left[\begin{array}{c} 0\\ \frac{I_{xeq}k_{1}}{mI_{xeq}-m_{s}^{2}h^{2}}\\ 0\\ 0 \end{array}\right]$$ (9)

In this paper, the active steering system is adopted based on the original tractor steering system. Between the steering wheel and the rack-and-pinion, planetary gear mechanism, power assisted motor and controller are installed additionally after cutting off the middle of the steering column of the original vehicle. In order to simplify the steering system design, motor-dominated steering model is obtained as follow.

$$G(s)=\frac{K}{T_{f}s^{2}+T_{e}s+1}$$ (10)

Where *K* is a constant relevant to the potential coefficient of the motor, *T*_*f*_ is the time constant of the armature circuit and the motor, and *T*_*e*_ is the electromechanical time constant of the motor.

### Model of tractor rollover indicator

Stability indicator is important for preventing tractor from rollover in the working process, it is the base of the rollover warning and active anti-rollover control. Different stability indicator design methods have been presented, such as Ding et al. proposed a comprehensive stability assessment scheme using tire fore estimation and load transfer ration value [24]. Song et al. proposed a simplified LTR indicator [25]. In this study, we define the rollover indicator using the load transfer ratio (LTR) which is denoted as the ratio of the difference between the vertical load of left and right wheels and the total tire load

$$LTR=\frac{F_{l}-F_{r}}{F_{r}+F_{l}}$$ (11)

Where *F*_*r*_, *F*_*l*_ are the vertical loads on the right and left wheels respectively, *F*_*r*_ = *F*_*l*_ = *mg*. A big absolute value of LTR indicates a higher possibility of rollover and vice versa. The value of LTR is related to the structural parameters and moving status parameters of tractor. Considering the actual working conditions of the tractor, a simplified description form of LTR is derived as follow.


$$LTR=\frac{2(k_{s}\phi +c_{s}\dot{\phi })}{mgd}$$ (12)

## Warning and control system design

### Structure of proposed anti-rollover control system

**Fig 3:**
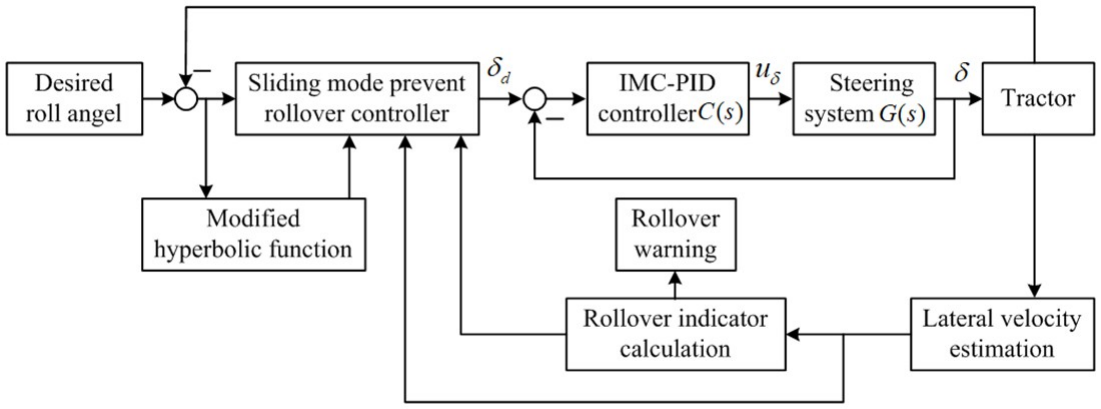
The overall architecture of the proposed control scheme.

As designed above, according to the calculated stability indicator, the control system can send out warning information to the driver or adjust the front wheel actively to prevent rollover. For this purpose, the overall architecture of the proposed scheme is demonstrated in Fig 3.

In the proposed control scheme, threshold values for the LTR are preset, such as *LTR*_*W*_ is used for rollover warning and *LTR*_*C*_ is used for rollover prevention control. When the calculated stability indicator is higher than *LTR*_*W*_, the system will activate the warning procedure as well as calculate the indicator constantly. If the calculated stability indicator is higher than *LTR*_*C*_, the sliding mode controller will play a part in preventing the tractor from rollover by active front wheel steering based on the roll angle control. What’s more, to reduce the chattering phenomenon, modified hyperbolic function is adopted to improve the performance of the sliding mode control. An IMC-PID steering controller is designed to track the desired front wheel obtained from the sliding mode controller. Furthermore, according to feedback correction principle, the lateral velocity is estimated using the difference between the measured lateral acceleration and estimated lateral acceleration from the dynamical model of the tractor.

### Lateral velocity estimation

As a state vector in the established tractor rollover model, the lateral velocity is an indispensable reference in tractor rollover control. Although some experts have put forward methods to measure the velocity directly by adopting sensors, but these methods are not applicable to engineering because the cost is high. Therefore, some methods for obtaining lateral or longitudinal velocity through estimation and multisensory fusion technique were proposed [26, 27]. In this study, a simple method based on the principle of feedback correction is adopted to estimate the lateral velocity. According to Newton’s second law and the torque balance equation, the state equation of the two degree of freedom model of the tractor is established as follows.

$$\left[\begin{array}{l} {\dot{V}}_{\text{y}}\\ \dot{r} \end{array}\right]=\left[\begin{array}{cc} a_{11} & a_{12}\\ a_{21} & a_{22} \end{array}\right]\left[\begin{array}{l} V_{y}\\ r \end{array}\right]+\left[\begin{array}{l} b_{11}\\ b_{\text{21}} \end{array}\right]\delta$$ (13)

where $a_{11}=\text{-}\frac{k_{1}+k_{2}}{mV_{x}}$, $a_{12}=\frac{-x_{1}k_{1}\text{+}x_{2}k_{2}}{mV_{x}}-V_{x}$, $b_{11}=\frac{k_{1}}{m}$, $a_{21}=\frac{-x_{1}k_{1}+x_{2}k_{2}}{I_{z}V_{x}}$, $a_{22}=-\frac{x_{1}^{2}k_{1}+x_{2}^{2}k_{2}}{I_{z}V_{x}}$, $b_{21}=\frac{x_{1}k_{1}}{I_{z}}$.

And the system output equation can be got as below.

$$\left[\begin{array}{l} a\\ r \end{array}\right]=\left[\begin{array}{cc} e_{11} & e_{12}\\ e_{21} & e_{22} \end{array}\right]\left[\begin{array}{l} V_{y}\\ r \end{array}\right]+\left[\begin{array}{l} f_{11}\\ f_{21} \end{array}\right]\delta$$ (14)

Where $e_{11}=\text{-}\frac{k_{1}+k_{2}}{mV_{x}}$, $e_{12}=\frac{-x_{1}k_{1}\text{+}x_{2}k_{2}}{mV_{x}}$, *e*_12_ = 0, *e* = 1, $f_{11}=\frac{k_{1}}{m}$, *f*_21_ = 0.

According to the tractor kinematics, the lateral velocity of the tractor can be estimated simply by the following formula.


$${\dot{\hat{V}}}_{y}=a-V_{x}r$$ (15)

The lateral velocity can be obtained by integrating the above equation about the measurable variables, such as lateral acceleration *α*, yaw velocity *r* and driving speed *V*_*x*_. In order to reduce the errors caused by sensors, a dynamic model is introduced to correct the estimation of lateral acceleration according to the feedback correction principle.

$${\hat{V}}_{y}=\int_{0}^{t}[(a-V_{x}r+k(a-\hat{a})]dt$$ (16)

Where *k* is the observer feedback gain, and $\hat{a}$ is the estimation of lateral acceleration according to the formula (14).


$$\hat{a}=-\frac{k_{1}\text{+}k_{2}}{mV_{x}}V_{y}+\frac{-x_{1}k_{1}\text{+}x_{2}k_{2}}{mV_{x}}r+\frac{k_{1}}{m}\delta$$ (17)

Considering the lateral acceleration of tractors is small in most field working environments, an adaptive feedback gain is designed to correct the accumulated error [28].


$$k=\frac{1}{\pi }\text{arctan}[10(\left|V_{x}r\right|-0.1)]-0.5$$ (18)

### Anti-rollover sliding mode controller design

As designed in the control scheme, the anti-rollover controller will be activated when the calculated stability indicator is higher than *LTR*_*C*_. Sliding control theory is adopted to design the prevent rollover controller, roll angle is taken as controlled variable and the output of the sliding mode controller is the desired front wheel angle. The roll angle error is defined as

$$e_{\phi }=\phi -\phi_{d}$$ (19)

Where *ϕ*_*d*_ is the desired roll angle according to the saturation function [29].


$$\phi_{d}\text{=}\left\{\begin{array}{c} 0.8\phi \begin{array}{cc} & \left|a\right|>0.35g \end{array}\\ \phi \begin{array}{cc} & \left|a\right|\leq 0.35g \end{array} \end{array}\right.$$ (20)

Traditional sliding surface with simple structure is selected as follow.

$$s=\eta e_{\phi }+{\dot{e}}_{\phi }=\eta \left(\phi -\phi_{d}\right)+\dot{\phi }-{\dot{\phi }}_{d}$$ (21)

Where, *η* is the sliding mode coefficient and *η* > 0, thus the derivative of the above equation is obtained as follow.

$$\dot{s}=\eta {\dot{e}}_{\phi }+{\ddot{e}}_{\phi }=\eta \left(\dot{\phi }-{\dot{\phi }}_{d}\right)+\ddot{\phi }-{\ddot{\phi }}_{d}$$ (22)

sgn function is selected as the approaching rate$\dot{s}=-\psi \text{sgn}\left(s\right)$. According to the established rollover model, we can get the following formula.


$$0.2\left[\eta \dot{\phi }+M_{1}V_{y}+M_{2}r+M_{3}\phi +M_{4}\dot{\phi }+M_{5}\delta_{\phi }\right]=-\psi \text{sgn}\left(s\right)$$ (23)

By solving the above equation, the desired front steering wheel can be obtained as follows.

$$\delta_{\phi }=\frac{1}{0.2M_{5}}\left[-\psi \text{sgn}\left(s\right)-0.2\left(\eta \dot{\phi }+M_{1}V_{y}+M_{2}r+M_{3}\phi +M_{4}\dot{\phi }\right)\right]$$ (24)

Where, $M_{1}=-\frac{(k_{1}+k_{2})m_{s}h}{(mI_{xeq}-m_{s}^{2}h^{2})V_{x}}$, $M_{2}=-\frac{(x_{1}k_{1}-x_{2}k_{2})m_{s}h}{(mI_{xeq}-m_{s}^{2}h^{2})V_{x}}$, $M_{3}=\frac{m(m_{s}gh-k_{s})}{mI_{xeq}-m_{s}^{2}h^{2}}$, $M_{4}=-\frac{mc_{s}}{mI_{xeq}-m_{s}^{2}h^{2}}$, $M_{5}=\frac{m_{s}hk_{1}}{mI_{xeq}-m_{s}^{2}h^{2}}$.

To avoid the chattering phenomenon caused by the sgn function in the output of the sliding mode controller and to protect the steering motor, we adopt an improved hyperbolic tangent function based sliding mode approaching rate function as

$$\text{sgn}(s)\text{=}\frac{1-e^{-us}}{1+e^{-us}}$$ (25)

The output of the prevent rollover controller based on the new approaching rate function can be obtained as

$$\delta_{\phi }\text{=}\frac{1}{0.2M_{5}}\left[-\psi \frac{1-e^{-us}}{1+e^{-us}}-0.2(\eta \dot{\phi }+M_{1}V_{y}+M_{2}r+M_{3}\phi +M_{4}\dot{\phi })\right]$$ (26)

### Front wheel angle controller design

The anti-rollover controller is designed to decrease the LTR by controlling the roll angle and its output is the desired front wheel angle. To track the desired steering angle, steering tracking controller is put forward to adjust the front steering wheel. As designed in the anti-rollover control system shown in Fig 3, the following equation can be obtained according to the design method of unit negative feedback controller based on internal model control theory.

$$G(s)K(s)=\frac{C(s)G(s)}{1+C(s)G(s)}$$ (27)

Where *G*(*s*) is the steering system, *C*(*s*)is the steering controller designed as a unit negative feedback controller, and *K*(*s*) is the internal model controller. For the steering system *G*(*s*) = *K*/(*T*_*f*_*s*^2^ + *T*_*e*_*s* + 1), the internal model controller is obtained as

$$K(s)=\frac{T_{f}s^{2}+T_{e}s+1}{K{\left(1+\lambda_{w}s\right)}^{2}}$$ (28)

Where λ_*w*_ is the adjust parameter for the controller. And the steering controller can be got as follow.


$$C(s)=\frac{K(s)}{1-G(s)K(s)}$$ (29)

To facilitate the engineering practice, the steering controller is designed in the PID form, and the output of the steering controller is

$$u_{\delta }(t)=K_{p}e_{\delta }(t)+K_{i}\int_{0}^{T}e_{\delta }(t)dt+K_{d}\frac{de_{\delta }(t)}{dt}$$ (30)

Where *e*_*δ*_(*t*) = *δ*_*d*_(*t*) − *δ*(*t*), *K*_*p*_ is the parameter of the proportional term, $K_{p}=T_{e}/(K\lambda_{w}^{2})$, *K*_*i*_ is the parameter of integral term, $K_{i}=1/(K\lambda_{w}^{2})$ and *K*_*d*_ is the parameter of differential term, $K_{d}=T_{f}/(K\lambda_{w}^{2})$.

## Simulation results

According to the proposed active anti-rollover control scheme, the controller should calculate the stability indicator in real time according to the parameters from the working state of the tractor. When the anti-rollover control condition is triggered, the control action can be implemented by adjusting the front steering wheel to control the LTR. In order to verify the effectiveness of the control strategy proposed in this paper, the simulation scheme is put forward as follows. A path is devised and a path tracking method is designed to track the path. During the travelling process, both the path tracking control and anti-rollover control are considered, but anti-rollover control action will play a leading role when the stability indicator is higher than *LTR*_*C*_. Because the keynote of this paper is studying on the anti-rollover control, the control of path tracking is not given in this paper. The path is produced by the following function

$$y_{d}=a_{1}\text{sin}\left(b_{1}t+c_{1}\right)+a_{2}\text{sin}\left(b_{2}t+c_{2}\right)+a_{3}\text{sin}\left(b_{3}t+c_{3}\right)$$

**Fig 4:**
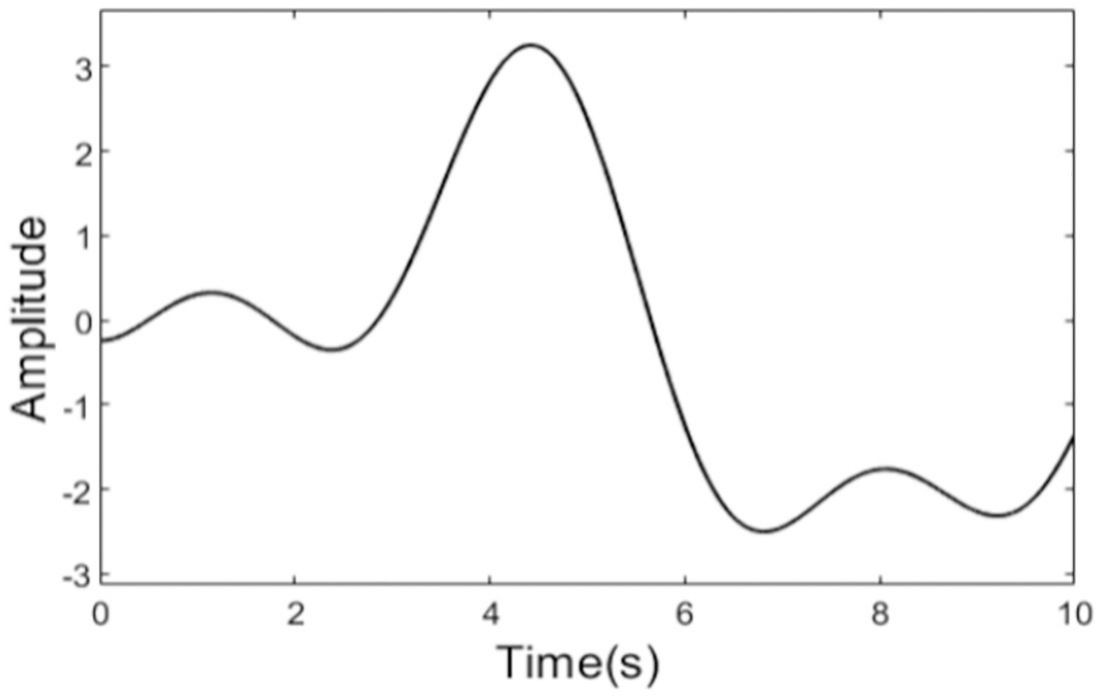
The designed path.

Where *a*_1_ = 30.5, *b*_1_ = 0.01022, *c*_1_ = 3.09, *a*_2_ = 2.012, *b*_2_ = 0.9315, *c*_2_ = 3.83, *a*_3_ = 1.086, *b*_3_ = 1.838, *c*_1_ = −0.5164, and the generated path is shown in Fig 4. Parameters of the tractor model and steering system model used in the simulation are shown in Table 1. Simulation models and controllers were established on Matlab/Simulink platform. In order to demonstrate the performance of the proposed control method, ordinary sliding mode control is also introduced and compared with the adaptive sliding mode control in the simulation. The parameters of the sliding mode controller are *η* = 2, *ψ* = 2, *u* = 5. The parameters of the steering system are *K* = 1.6368, *T*_*f*_ = 0.16, *T*_*e*_ = 0.56, *λ*_*w*_ = 0.6. As a parameter in the model, speed of the tractor is set as 37.5km/h in the simulation.

**Table 1:** Tractor parameters.

Parameter	Unit	Value
Mass *m* Sprung mass *m*_*s*_ Moment of inertia around the roll center *I*_*xeq*_	Kg Kg kg·m^2^	2700 2100 1493
Moment of inertia around the Z axis *I*_*z*_	kg·m^2^	3600
Wheelbase *L*	m	1.5
Cornering stiffness of the front wheel *k*_1_	KN·rad^-1^	351917
Cornering stiffness of the rear wheel *k*_2_ Roll damping of the suspension *C*_*s*_ Roll stiffness of the suspension *K*_*s*_ Distance from the center of mass to front axis *x*_1_ Distance from the center of mass to rear axis *x*_2_	KN·rad^-1^ N·s/m N/m m m	366466 6388 727811 1.212 0.976
Distance from the center of mass to roll center h Tread d	m m	0.6 0.7

**Fig 5:**
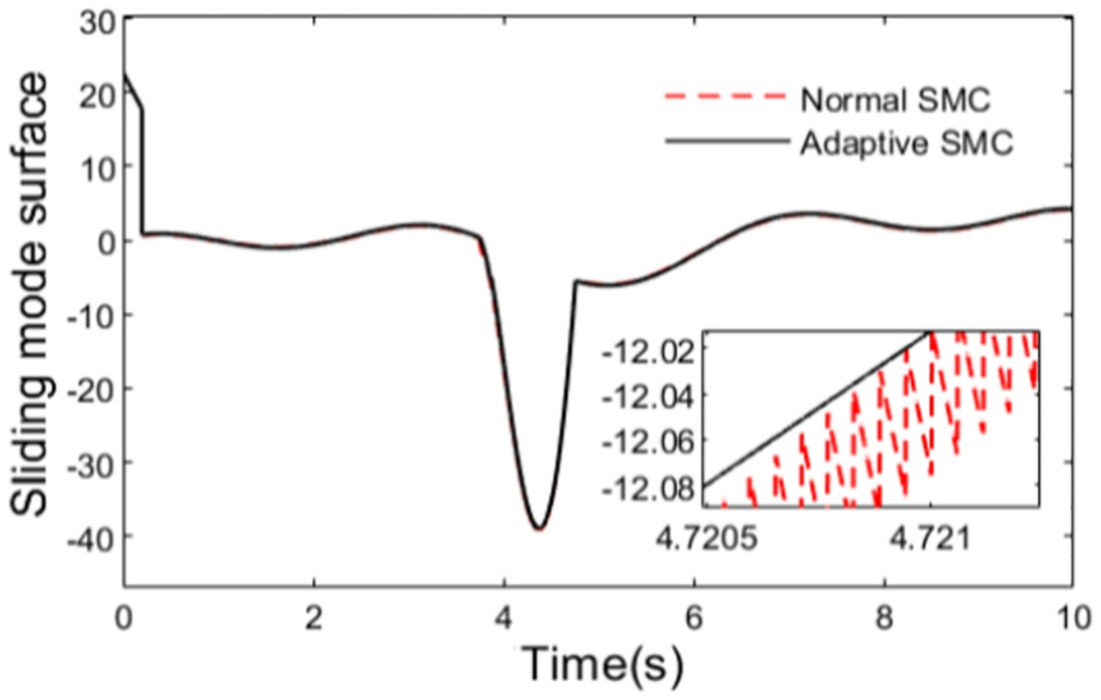
Sliding mode surface.

Simulation is performed from the starting point of the planed path based on the established tractor model and the designed controllers. The sliding mode surfaces for the two anti-rollover controllers are shown in Fig 5. It can be seen from the figure that the sliding mode surface of the proposed simple hyperbolic function is smoother than that of the conventional sliding mode controller, which can reduce the chattering phenomenon to a certain extent in the process of control.

**Fig 6:**
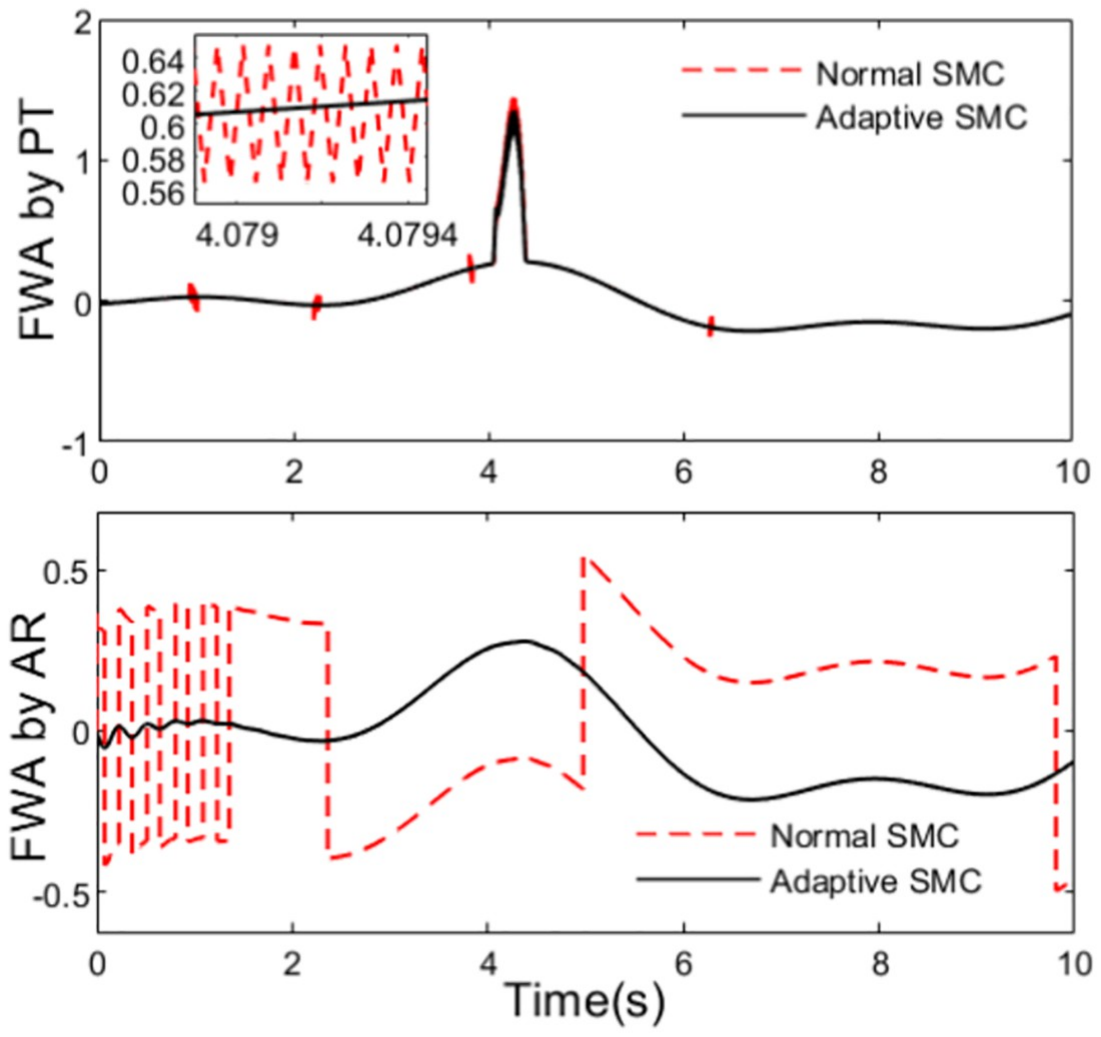
Desired front wheel angle.

As designed above, anti-rollover controller calculates the rollover indicator LTR in real time during the traveling of the tractor. In this section, *LTR*_*C*_ = 0.8 is set. In the simulation process, both path tracking controller and anti-rollover controller output the desired front steering wheel, but desired front steering wheel obtained by anti-rollover controller is adopted only the LTR is bigger than 0.8. For the planed path, the front wheel angles obtained from the path tracking controller and the anti-rollover controller are shown in Fig 6.

**Fig 7:**
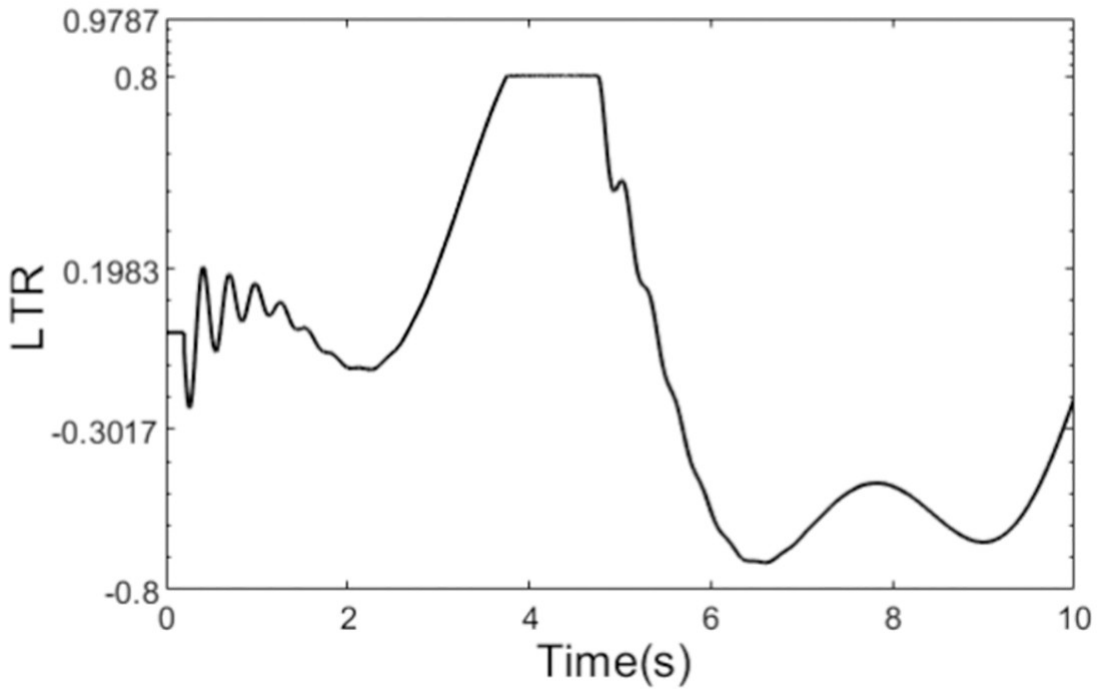
Real time value of LTR.

**Fig 8:**
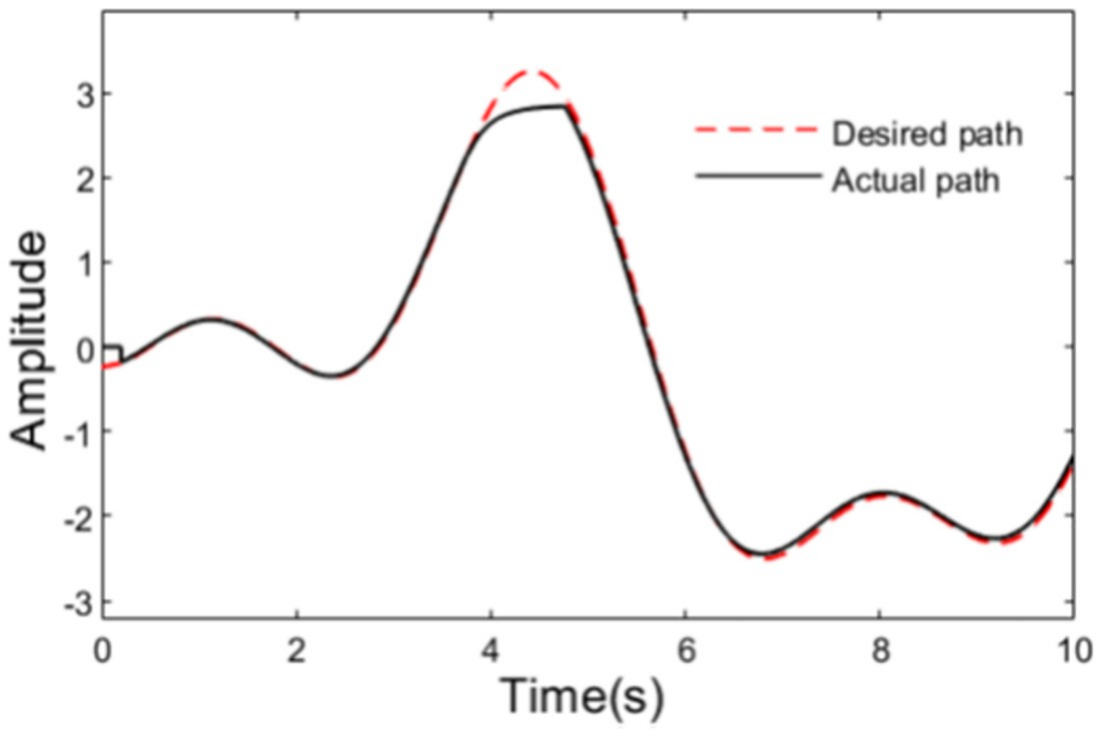
Desired and actual path.

As can be seen from the Fig 4 that there is a sharp curve in the path at t = 4s, and there is a danger of rollover for the tractor. At the same time, we can see from Fig 6 that there are great changes in the outputs of the two controllers at t = 4s. Obviously, to track the sharp curve, the great change in the output of path tracking controller is inevitable. Fig 6 illustrates clearly that there is serious chattering in the output of anti-rollover controller based on the conventional sliding mode control, while the output of the controller designed using adaptive sliding mode control is very smooth. What’s more, the graph of calculated rollover indicator LTR is shown in Fig 7, from which we can see that LTR reaches 0.8 about t = 4s. At this moment, desired front steering wheel is decided by the anti-rollover controller, and path tracking controller does not take effect until the LTR is controlled within safe limit. So in order to prevent the tractor from rollover as far as possible, the control action of the anti-rollover controller may drive the tractor to deviate from the plan path. The planed path and the actual travelling path are shown in Fig 8.

**Fig 9:**
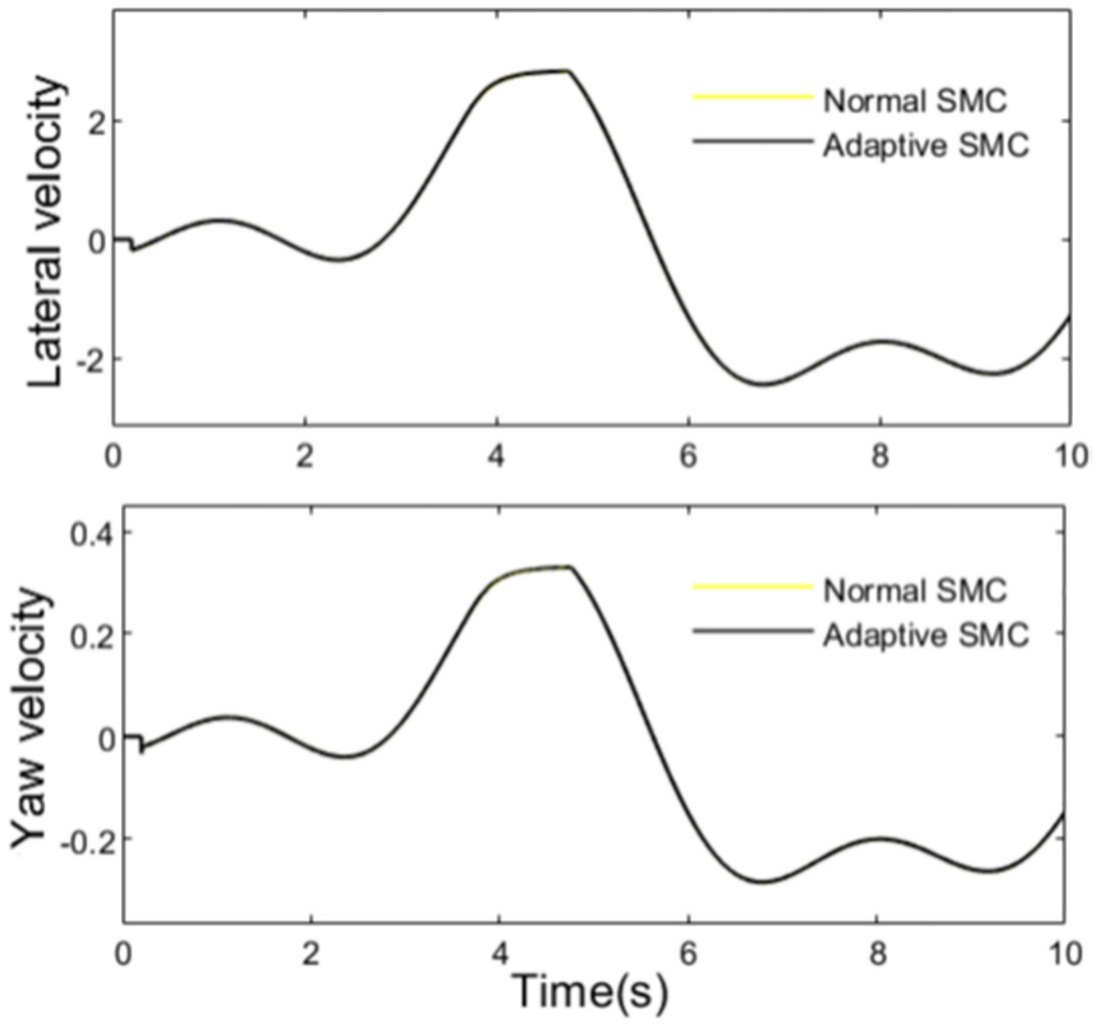
Lateral velocity and yaw velocity.

**Fig 10:**
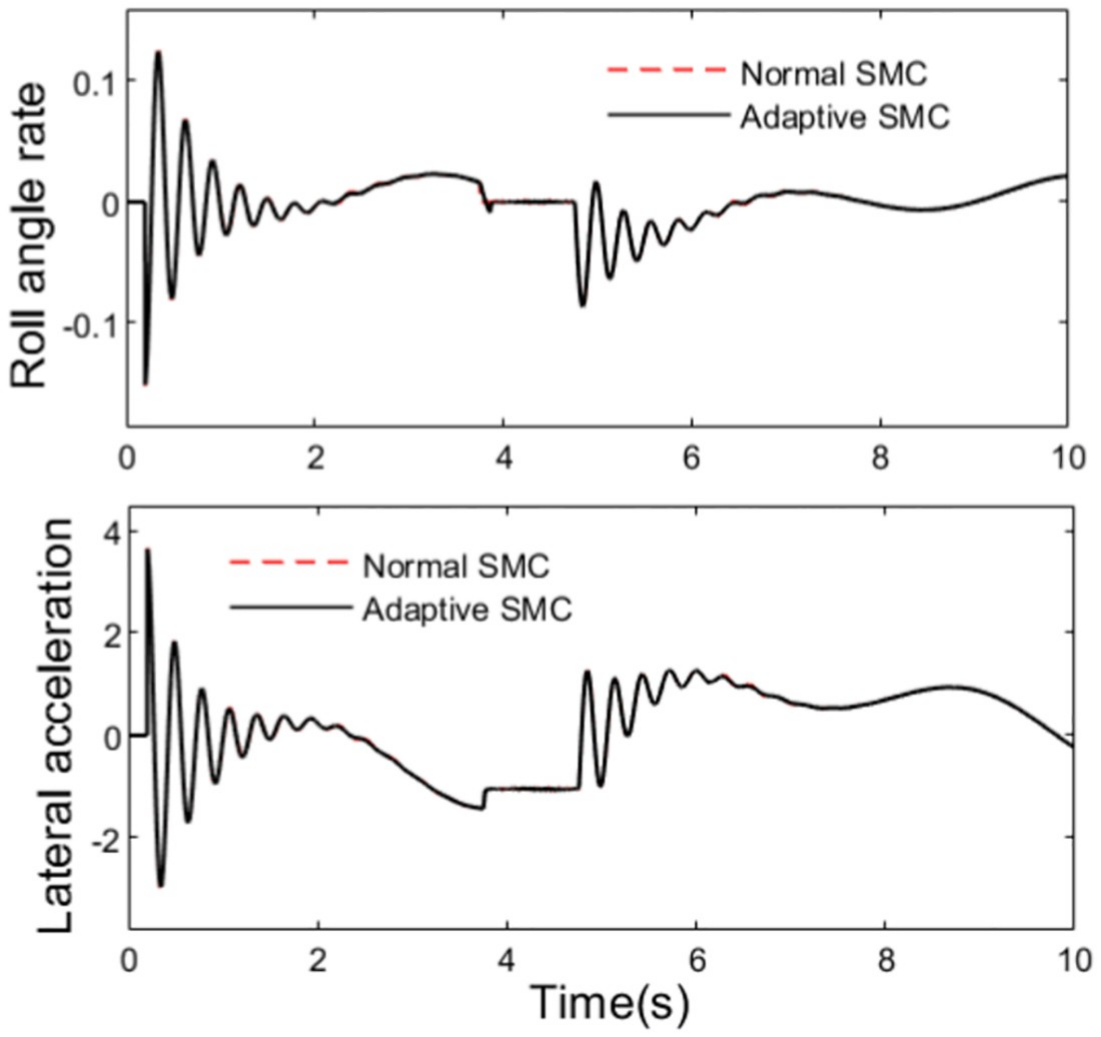
Roll angle rate and lateral acceleration.

State parameters of the travelling tractor are shown in Figs 9 and 10. It can be seen from the figures that the trend of the lateral velocity, yaw velocity, lateral acceleration and roll angle of the tractor are in accord with the change of the path, and they are controlled by the anti-rollover controller when the rollover indicator LTR exceeds the set value *LTR*_*C*_.

## Conclusions

Among all incidents along with agricultural mechanization, tractor rollover is the leading cause of fatalities. In this paper, anti-rollover warning and active control was studied based on the front wheel steering control technique. A state equation describing the tractor rollover dynamic properties was established with the front wheel angle as control variables. A simplified LTR model of tire was presented to evaluate the risk of rollover. Automatic steering system model including motor and mechanical steering mechanism was developed. The lateral velocity of the tractor was estimated by introducing an adaptive feedback gain according to the deviation between the measured lateral acceleration value and the lateral acceleration value estimated based on the dynamic model. The roll angle control used to prevent rollover was designed based on the TDOF rollover dynamic model and sliding mode control theory. As output of the sliding mode controller, the desired front wheel angle was tracked by an IMC-PID controller designed according to the established automatic steering system model. Simulations were carried out by navigating the tractor on the designed path while the anti-rollover control played a leading role when the rollover indicator was higher than the preset value. The simulation results demonstrated that the proposed method could control the LTR effectively and prevent the tractor from rollover.

## References

[1] Rondelli V, Casazza C, Martelli R. Tractor rollover fatalities, analyzing accident scenario. J Safety Res. 2018;67:99–106. doi: 10.1016/j.jsr.2018.09.015 30553435

[2] Abubakar MS, Ahmad D, Akande FB. A review of farm tractor overturning accidents and safety. Pertanika J Sci Tech. 2010;18(2):377–385. http://psasir.upm.edu.my/id/eprint/10395/1/27.pdf.

[3] Kim UK, Rehkugler GE. A review of tractor dynamics and stability. Transactions of the ASAE.2013;30(3):615–623. doi: 10.13031/2013.30449

[4] Ahmadi I. Dynamics of tractor lateral overturn on slopes under the influence of position disturbances (model development). J Terrammechanics.2011;48(5):339–346. doi: 10.1016/j.jterra.2011.07.001

[5] Guzzomi AL, Rondelli V, Guarnieri A, Molari G, Molari PG. Available energy in the rollover of narrow track wheeled agricultural tractors. Biosyst Eng.2009;104(3):318–323. doi: 10.1016/j.biosystemseng.2009.07.005

[6] Silleli H, Dayioglu M A, Gultekin A, Saranli G, Yildiz MA, Akay E, et al. Anchor mechanism to increase the operator clearance zone on narrow-track wheeled agricultural tractors: static and field upset test results. Biosyst Eng.2008;99(2):196–204.

[7] Ahmadi I. Development of a tractor dynamic stability index calculator utilizing some tractor specifications. Turk J Agric For.2013;7(2):203–211. doi: 10.3906/sag-1211-60

[8] Franceschetti B, Lenain R, Rondelli V. Comparison between a rollover tractor dynamic model and actual lateral tests. Biosyst Eng. 2014;127:79–91.

[9] Li Z, Mitsuoka M, Inoue E, Okayasu. Dynamic analysis of agricultural wheel tractor driving on uneven surface under the influences of speed and slope angle. J Fac Agr Kyushu U. 2014;59(2): 339–343. doi: 10.5109/1467644

[10] Guzzomi A L. A revised kineto-static model for Phase I tractor rollover. Biosyst Eng. 2012;113(1):65–75. doi: 10.1016/j.biosystemseng.2012.06.007

[11] Li Z, Mitsuoka M, Inoue E, Okayasu, Hirai Y. Development of stability indicators for dynamic Phase I overturn of conventional farm tractors with front axle pivot. Biosyst Eng. 2015;134:55–67. doi: 10.1016/j.biosystemseng.2015.03.016

[12] Baker V, Guzzomi AL. A model and comparison of 4-wheel-drive fixed-chassis tractor rollover during Phase I. Biosyst Eng. 2013; 116(2):179–189.

[13] Jang M K, Hwang S J, Kim J H, Nam J S. Overturning and rollover characteristics of a tractor through dynamic simulations: Effect of slope angle and obstacles on a hard surface. Biosyst Eng. 2022;219:11–24. doi: 10.1016/j.biosystemseng.2022.04.017

[14] Franceschetti B, Rondelli V, Ciuffoli A. Comparing the influence of Roll-Over Protective Structure type on tractor lateral stability. Safety Sci. 2019;115:42–50. doi: 10.1016/j.ssci.2019.01.028

[15] Sun C, Nakashima H, Shimizu H, Miyasaka J, Ohdoi K. Physics engine application to overturning dynamics analysis on banks and uniform slopes for an agricultural tractor with a rollover protective structure. Biosyst Eng. 2018; 28(9):1–11.

[16] Chou HY, Khorsandi F, Vougioukas SG, Fathallah FA. Developing and evaluating an autonomous agricultural all-terrain vehicle for field experimental rollover simulations. Comput Electron Agr. 2022;194(3):106735. doi: 10.1016/j.compag.2022.106735

[17] Guzzomi AL, Rondelli V, Capacci E. Operator protection in rollover events of articulated narrow track tractors. Biosyst Eng. 2019;185(9):103–115.

[18] Wu J, Kong Q, Yang K, Liu Y, Cao, Li Z. Research on the steering torque control for intelligent vehicles Co-Driving with the penalty factor of human-machine intervention. IEEE T Syst Man Cy-s.

[19] Liu B, Bulent KA. Safe Driving: A mobile application for tractor rollover detection and emergency reporting. Comput Electron Agr. 2013; 98:117–120.

[20] Qin J H, Zhu Z H, Ji H Y, Zhu Z X, Li Z, Du Y F, et al. Simulation of active steering control for the prevention of tractor dynamic rollover on random road surfaces. Biosyst Eng. 2019;185:135–149. doi: 10.1016/j.biosystemseng.2019.02.006

[21] Qin J H, Wu A B, Song Z S, He Z Z, Suh C S, Zhu Z X, et al. Recovering tractor stability from an intensive rollover with a momentum flywheel and active steering: System formulation and scale-model verification. Comput Electron Agr.2021;190(11):106458. doi: 10.1016/j.compag.2021.106458

[22] Song Z S, Wang L L, Liu Y M, Wang K D, He Z Z, Zhu Z X, et al. Actively steering a wheeled tractor against potential rollover using a sliding-mode control algorithm: Scaled physical test. Biosyst Eng. 2022;213:13–29. doi: 10.1016/j.biosystemseng.2021.11.015

[23] Wu J, Zhang J, Nie B, Liu Y, He X. Adaptive control of PMSM servo system for steering-by-wire system with disturbances observation. IEEE T Transp Electr. 2022; 8(2):2015–2028.

[24] Ding XL, Wang ZP, Zhang L, Liu JZ. A Comprehensive vehicle stability assessment system based on enabling tire force estimation. IEEE T Veh Technol. 2022, 1–17.

[25] Song YL, Zhang XH, Wang W. Rollover dynamics modelling and analysis of self-propelled combine harvester. Biosyst Eng. 2021;209(7):271–281.

[26] Ding X L, Wang Z P, Zhang L. Event-triggered vehicle sideslip angle estimation based on low-cost sensors. IEEE T Ind Inform. 2022;18(7):4466–4476.

[27] Ding XL, Wang ZP, Zhang L, Wang C. Longitudinal vehicle speed estimation for four-wheel-independently-actuated electric vehicles based on multi-sensor fusion. IEEE T Veh Technol. 2020;69(11):12797–12806. doi: 10.1109/TVT.2020.3026106

[28] Limroth J. Real-time vehicle parameter estimation and adaptive stability control, Clemson University, 2009. https://tigerprints.clemson.edu/all_dissertations/494.

[29] Zhang L. Anti-rollover control of vehicle based on chassis dynamics, Nanjing University of Aeronautics and Astronautics, 2016. https://d.wanfangdata.com.cn/thesis/ChJUaGVzaXNOZXdTMjAyMjA1MjYSCUQwMTM4Mzg1MBoIY3J3b3Z3Z3U%3D.

